# Histomorphological transformation from non-small cell lung carcinoma to small cell lung carcinoma after targeted therapy or immunotherapy: A report of two cases

**DOI:** 10.3389/fonc.2022.1022705

**Published:** 2022-11-09

**Authors:** Hao Liu, Li-Hong Chen, Zhi-Hui Zhang, Ning Wang, Si-Hui Zhuang, Hao Chen, Jin Du, Li-Juan Pang, Yan Qi

**Affiliations:** ^1^ Department of Pathology, Shihezi University School of Medicine & the First Affiliated Hospital to Shihezi University School of Medicine, Shihezi, Xinjiang, China; ^2^ Department of Pathology, Zhanjiang Central Hospital, Guangdong Medical University, Zhanjiang, China; ^3^ Department of Thoracic Surgery, Zhanjiang Central Hospital, Guangdong Medical University, Zhanjiang, Guangdong, China

**Keywords:** lung cancer, EGFR-TKI, PD-1 inhibitors, histomorphological transformation, targeted therapy, immunotherapy

## Abstract

Molecular targeting and immunotherapy provide durable responses for advanced lung cancer clinical therapy in many patients. However, the mechanisms of occurrence of progressive disease and resistance to targeted therapy and immunotherapy have not been elucidated. Herein, we report two cases of small cell transformation of non-small cell lung cancer (NSCLC) after targeted therapy or immunotherapy. The first case was a 63-year-old female patient presenting with cough and expectoration. Left lung invasive adenocarcinoma was diagnosed after left lung tumor biopsy. After epidermal growth factor receptor-tyrosine kinase inhibitor (EGFR-TKI) targeted therapy for almost 2 years, disease progression and symptom aggravation were observed. Pathological and immunohistochemical staining results after biopsy revealed small cell lung cancer (SCLC). The second case was a 75-year-old male patient diagnosed with stage IV squamous cell carcinoma of the lung, who received carboplatin/paclitaxel adjuvant chemotherapy and pembrolizumab treatment with partial response. Disease progression and metastasis occurred within 15 cycles of immunotherapy. Computed tomography revealed a lower left lung tumor. Cytological examination of lung lavage fluid and biopsy under thoracoscope revealed SCLC. In conclusion, histological transformation to SCLC is a potential mechanism of NSCLC resistance to targeted therapy or immunotherapy. During treatment, clinicians should monitor serum tumor markers or genome sequencing, particularly in patients with disease progression, as this may be beneficial for early detection of SCLC transformation. Repeated biopsy can be performed if necessary, and the therapeutic regimen can be adjusted in a timely manner according to the results of molecular pathological tests for personalization and whole-process management.

## Introduction

Lung cancer is the most commonly diagnosed cancer and is the leading cause of cancer-related deaths (1). The World Health Organization (WHO) classifies lung cancer into two broad histological subtypes: non-small-cell lung cancer (NSCLC), which underscores 85% of cases, and small-cell lung cancer (SCLC), which accounts for the remaining 15%. NSCLC is further subdivided into adenocarcinoma, squamous-cell carcinoma, and large-cell carcinoma (2). Individualized treatment of lung cancer is crucial, and the therapeutic regimens for different subtypes and stages are distinct. The optimal strategy of treatment for stage I and II NSCLC is radical resection, and patients with high recurrence risk may receive combined postoperative radiotherapy (3). For unresectable locally advanced NSCLC, the standard treatment is immunotherapy and concurrent chemoradiation (4). In patients with SCLC, systemic treatment is required as the main or auxiliary treatment, even in the era of limited imaging. For patients with limited-stage SCLC, the goal of treatment is to be cured by chemotherapy and chest radiotherapy. Patients who are fit to undergo radical surgery should after evaluation be administered systemic treatment after surgery and selective mediastinal radiotherapy. Chemotherapy combined with immunotherapy is preferred in patients with extensive-stage SCLC. In addition, systemic therapy alone can alleviate symptoms and appropriately prolong survival in most patients, but long-term survival is generally rare (5). Epidermal growth factor receptor (*EGFR*) is one of the most common driving genes in NSCLC. Progress in molecular biology research has resulted in EGFR-tyrosine kinase inhibitors (EGFR-TKIs) emerging as the first-line treatment for patients with EGFR-mutant cancer. However, the majority of patients inevitably develop cross-acquired resistance after 9-13 months of treatment with first- and second-generation EGFR-TKIs through various mechanisms (6). The several mechanisms are associated with the development of acquired resistance to EGFR-TKIs, include T790M mutation, c-MET amplification, *KRAS* mutation, *BIM* polymorphism deletion, and *PIK3CA* gene mutations (7). The mechanism of EGFR-TKI resistance and treatment strategies after resistance have become the focus of basic and clinical research. Recently, a growing number of drugs targeting tumor drug resistance mechanisms and related signaling pathways have entered clinical trials. This has resulted in development of immunotherapy and widespread use of immune checkpoint inhibitors (ICI) for the treatment of patients with lung cancer. The 2020 update of the NCCN Oncology Clinical Practice Guidelines (NCCN Guidelines) recommends new first-line immunotherapy regimens for NSCLC, including pembrolizumab monotherapy, pembrolizumab in combination with chemotherapy, and atrizumab or bevacizumab in combination with chemotherapy (8). For example, the representative drug, pembrolizumab, is a highly selective humanized anti-PD-1 IgG4κ monoclonal antibody. Disrupting the binding of programmed death-1 (PD-1) and its ligand, the key immunomodulatory factor programmed cell death ligand 1 (PD-L1), blocks inhibitory signals in T cells and inhibits the CD8 cytotoxic immune response and resulting anti-tumor immune response (9, 10). Of note, a subset of patients treated with ICIs, such as PD-L1 inhibitors/PD-1 inhibitors, also exhibittransformation to SCLC. In this regard, the transformation of NSCLC to SCLC may constitute a mechanism of action of anti-PD-1/PD-L1 inhibitors.

A minority of patients present with small cell histomorphological transformation of NSCLC, including adenocarcinoma or squamous cell carcinoma, after targeted therapy or immunotherapy. However, transformation to SCLC is considered drug resistance, and the underlying mechanisms remain unclear (11, 12). The transformation of NSCLC to SCLC after targeted therapy or treatment with ICIs alone or in combination with other drugs as anti-tumor therapy has been reported (13–15). Here, we report a case of TKI resistance due to SCLC transformation and another case of tumor transformation from squamous cell carcinoma to SCLC during treatment with pembrolizumab. Our cases emphasize the importance of repeated biopsy in the treatment process and propose a possible source of transformed cells. In addition, we provide a review of the literature and summarize the clinical characteristics of rare SCLC transformation cases with PD-1 inhibitors (12, 15–23).

## Case description

### Case 1

**Figure 1:**
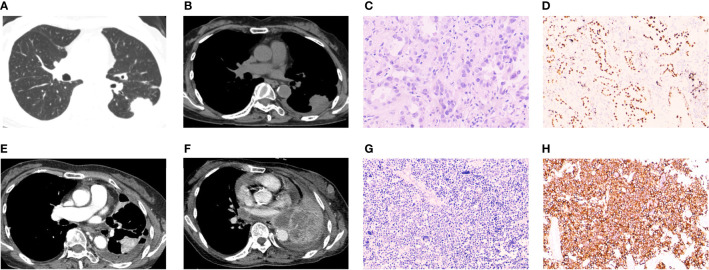
Radiographic and pathological images of Case 1. **(A, B)** A computed tomography (CT) scan at first visit showing a left lung mass with lung window and mediastinum window. **(C)** Biopsy specimens of the left lung mass revealed malignant cells with adenoid structure, abundant cytoplasm, eosinophilic, and round nuclei, suggesting infiltrating adenocarcinoma of the left lung (H&E staining, ×200 magnification). **(D)** Immunohistochemical (IHC) staining revealed transcription termination factor 1 (TTF-1) immunopositivity (TTF-1 staining, × 100 magnification). **(E)** CT scan showing progression of left pulmonary lesions after 14 months of gefitinib use. **(F)** CT scan showed multiple nodules and masses in the left lung. The lesions were increased, enlarged and fused compared with 5 months ago. Multiple enlarged lymph nodes were seen, and multiple metastasis was considered. **(G)** Biopsy after mass resection revealed malignant cells, which were small, round, and arranged in small nests, with minimal cytoplasm and hyperchromic nuclei (H&E staining, ×200 magnification). **(H)** IHC staining revealed diffuse strong positive expression of CD56 in cancer cells (CD56 staining, ×100 magnification). Morphology and immunohistochemistry were consistent with small cell lung cancer.

A 63-year-old woman with no smoking history was admitted for cough and expectoration in June 2020. A computed tomography (CT) scan revealed a 3.3×2.5 cm mass in the lower left lung and multiple mediastinal lymph nodes (**Figures 1A, B**). After percutaneous puncture lung biopsy, the removed tissues were diagnosed pathologically by immunohistochemistry (IHC) and hematoxylin-eosin (H&E) staining (**Figure 1C**). IHC staining results indicated that both CK7 and napsin A were expressed in the cytoplasm and were strongly positive, while thyroid transcription factor 1 (TTF-1) was expressed in the nucleus and was positive (**Figure 1D**). The positive index of Ki-67 was 5%. The diagnosis of left lung invasive adenocarcinoma was considered based on the combined imaging and pathological biopsy results. The genetic testing results revealed *EGFR* mutation, and targeted therapy with EGFR-TKIs was recommended. The patient was commenced on gefitinib. At 5 months after chemotherapy, the patient presented with dry cough and chest tightness 1 week prior in November 2020. After admission, CT revealed malignant pleural effusion. Routine pleural effusion estimation results of carcinoembryonic antigen (CEA) level showed that they were abnormal and increased. By careful consultation, the patient had discontinued gefitinib for 5 months and switched to traditional Chinese medicine. By dialogue, resumption of targeted therapy usage with gefitinib was suggested. In August 2021, the patient presented with chest tightness and shortness of breath. CT scan results that were compared with previous 9-month images revealed partial enlargement of the left lung lesion and a large pleural effusion on the left side (**Figure 1E**). After symptom relief by pleural effusion drainage, the patient underwent thoracic perfusion. The patient’s symptoms and examination results indicated progressive adenocarcinoma of the left lung that was resistant to gefitinib. The use of third-generation targeted drugs or combined chemotherapy for follow-up treatment after completion of genetic detection of drug resistance in lung cancer was suggested, but this was rejected by the patient. In February 2022, the patient again presented with chest tightness, shortness of breath, and aggravation after activity. Enlarged lymph nodes with hard texture were palpable in the anterior left neck and outer quadrant of the left breast, with poor activity. Review of the chest CT compared to those obtained in August 2021 revealed progression of the lesion in the left lung, with multiple lymph node and organ metastases (**Figure 1F**). Biopsy was performed after the resection of a 2.0×1.5 cm tumor section on the left chest wall (**Figure 1G**). IHC staining revealed strong diffuse synaptophysin (Syn) immunopositivity in the cytoplasm, focal weak CD56 immunopositivity in the cell membrane (**Figure 1H**), and TTF-1 immunopositivity in the nucleus. The positive index of Ki-67 was 90%, and the patient was diagnosed with SCLC. Tumor markers, tested severally during the patient’s multiple hospitalizations revealed abnormally high CEA levels. Other indicators (CA199, human epithelial protein 4 (HE4), and ferritin) levels were also abnormal, and the last reexamination of serum tumor markers revealed significantly elevated neuron-specific enolase (NSE) levels to 159.00 ng/mL (normal range, 5-15 ng/mL). Suggested continuation of subsequent SCLC chemotherapy was rejected by the patient and her family. Unfortunately, due to progressive disease (PD), the patient eventually died in March 2022 due to respiratory failure caused by acute respiratory distress syndrome (ARDS) combined with acute right pulmonary edema.

### Case 2

**Figure 2:**
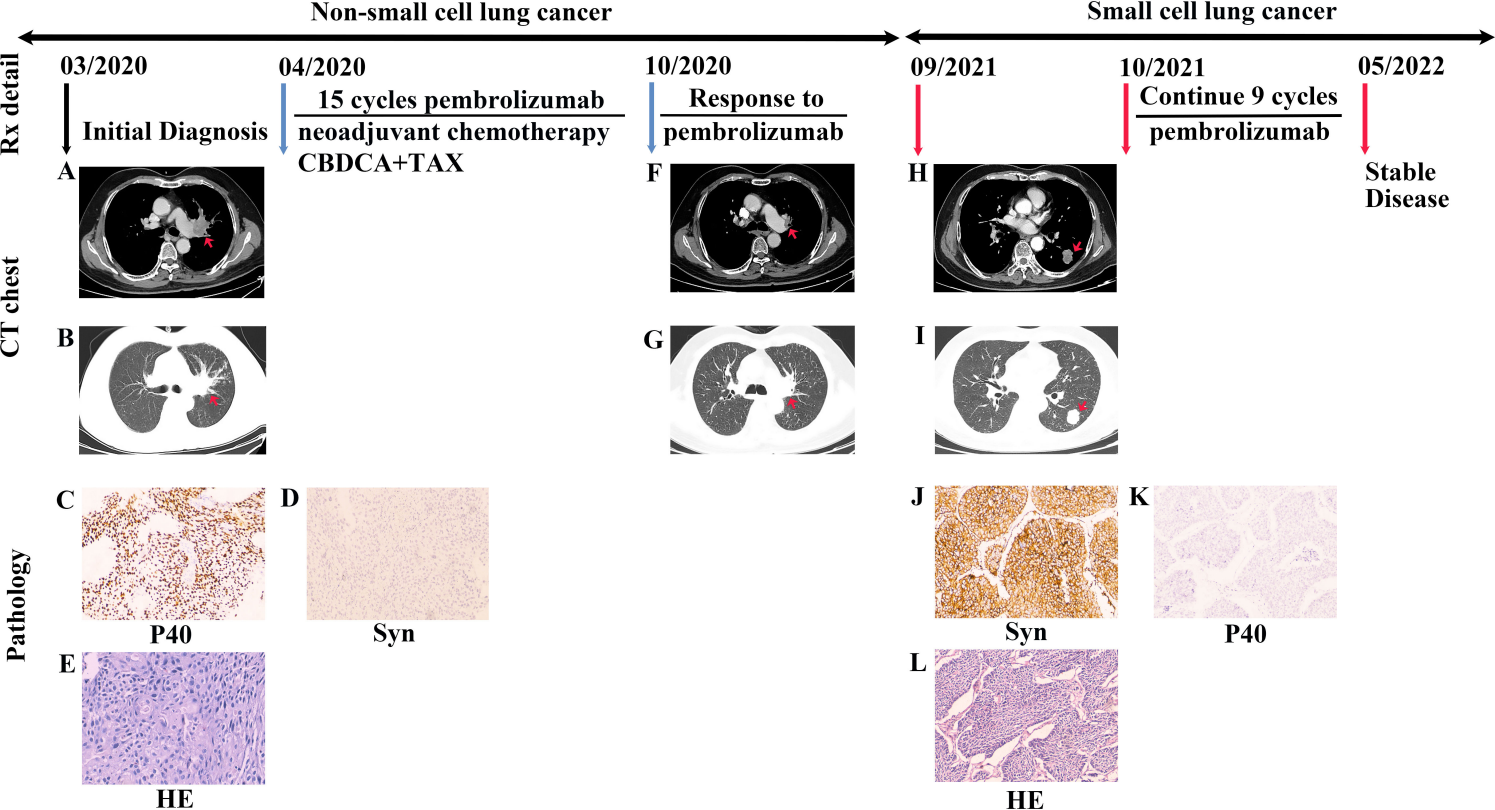
Case presentation of transformation of lung squamous cell carcinomas to small cell lung carcinoma with pembrolizumab. **(A, B)** Chest CT scan showing a space-occupying lesion of the left hilar with mediastinum window and lung window. **(C, D)** Biopsied specimens subjected to IHC staining showed that P40 was strongly positive, and Syn was negative (P40 and Syn staining, ×100 magnification). **(E)** Based on resected specimens, the pathological diagnosis was lung squamous cell carcinoma (H&E staining, ×200 magnification). **(F, G)** The patient was treated with a regimen of ICIs combined with chemotherapy and exhibited a partial response. The lesion was significantly reduced in the mediastinum window and lung window than before treatment. **(H, I)** After completing 15 cycles of immunotherapy, CT scans revealed tumor progression and new lesions in the left lower lung in the mediastinum window and lung window. **(J, K)** IHC staining revealed strong Syn immunopositivity in the cytoplasm and negative staining for P40 (Syn and P40 staining, ×100 magnification). **(L)** Biopsy resulted in tumor diagnosis of small cell lung cancer (H&E staining, ×100 magnification). Rx, treatment; CT, computed tomography; H&E, hematoxylin and eosin; CBDCA, carboplatin; TAX, paclitaxel; Syn, synaptophysin.

A 75-year-old Chinese man with a smoking history of 40 pack-years presented with cough. A CT chest scan confirmed a space-occupying lesion on the left hilar and mediastinal lymph node enlargement in March 2020 (**Figures 2A, B**). Fiberoptic bronchoscopy revealed complete blockage of the upper lobe opening of the left lung. The specimens were biopsied under bronchoscopic guidance with strong positive IHC staining for P40 (**Figure 2C**) and P63 in the nucleus, and positive expression of CK5/6 and CK7 in the cytoplasm. The Ki-67 positive index was 60%. Specimens were negative for TTF-1 in the nucleus and for napsin-A and Syn (**Figure 2D**) in the cytoplasm. Even cornified pearls were observed in H&E sections (**Figure 2E**). The patient was diagnosed with left central squamous cell carcinoma with multiple metastases to both lungs . An adjuvant chemotherapy regimen of carboplatin-paclitaxel was administered and the first cycle of a PD-1 inhibitor, pembrolizumab, was commenced at a dose of 200 mg every 3 weeks from April 2020. The patient was treated for NSCLC with ICIs combined with chemotherapy and exhibited a partial response (**Figures 2F, G**). The patient completed 15 cycles of immunotherapy by September 2021. Compared to the CT findings in May 2021, a follow-up CT scan revealed tumor progression, metastases, and new lesions in the left lower lung (**Figures 2H, I**). Serum CEA levels were slightly high (9.27 ng/mL). Referring to the patient’s imaging data, clinicians considered the new lesions to be oligoprogression within an oligometastatic clinical situation. Surgical indications were evident, and the patient underwent neoplasm excision by thoracoscopy, with a 3.0 x 2.5 cm mass in the left lower lobe. Pathological biopsy by surgical excision of the specimen and IHC staining revealed weak focal immunopositivity for CD56, diffuse TTF-1 immunopositivity in the nucleus, and strong immunopositivity for Syn (**Figure 2J**) and CK7 in the cytoplasm. The Ki-67 positive index was 40%. Staining for P40 (**Figure 2K**), P63, napsin-A, and CK5/6 was negative. Biopsy revealed consistent characteristics with SCLC (**Figure 2L**). Gene sequencing results indicated the absence of *EGFR* mutation and ALK translocation. The patient was advised to continue follow-up treatment with ICIs. Immunotherapy with pembrolizumab was commenced in October 2021. As of September 2022, 20 cycles of treatment had been completed, with good clinical outcomes and no significant discomfort.

## Discussion

In this report, we describe small cell histomorphological transformation of NSCLC after immunotherapy (Case 1) and EFGR-TKI targeted (Case 2) therapy. The common features of both cases were as follows: (1) before the transformation to SCLC, the lesions in both cases were multicentric and imaging results revealed enlarged lymph nodes; and (2) transformation to neuroendocrine SCLC occurred in both cases. Moreover, differences between the two cases were as follows: (1) the patients were of different gender and the male patient had a long history of smoking; (2) the histological types prior to transformation were distinct, whereby Case 1 was lung adenocarcinoma and Case 2 was squamous cell lung carcinoma; (3) different driving genes and molecular changes led to the use of different therapeutic drugs, whereby EGFR-TKI was used for Case 1 and a PD-1 inhibitor was used for Case 2; (4) the patients exhibited distinct therapeutic effects. Case 1 was not sensitive to targeted therapy and showed PD, while Case 2 was sensitive to immunotherapy and stable disease (SD).

Targeted therapy with EGFR-TKIs is often the first-line treatment for lung cancers with *EGFR*-activating mutations. The most common resistance mechanism is a Thr790Met mutation in *EGFR*, which has been reported in 50-60% of resistant tumor samples (24). *MET* and *HER2* amplify at low frequencies, accounting for another 15-20% of cases. Histological transformation of *EGFR*-mutated adenocarcinoma to SCLC was first reported in 2006 as a relatively rare mechanism of resistance (2). Among patients treated with EGFR-TKIs as targeted therapy, this mechanism accounts for 3-15% of transformation to SCLC, particularly transformation from adenocarcinoma to SCLC (25), which may constitute a potential mechanism of acquired drug resistance to EGFR-TKIs (26). Small cell transformation as a resistance mechanism for immunotherapy was first reported in 2017 by Imakita et al. (23). Since then, several similar cases have been reported, and the concept of small cell transformation is well known.

Epithelial-mesenchymal transformation (EMT) has been proposed as a mechanism of acquired resistance to EGFR-TKIs in several preclinical models. Deficiency in apoptosis leads to resistance of EGFR-TKIs in NSCLC with *EGFR*-mutation, and a reduction in apoptosis is often caused by the lack of expression of the pro-apoptotic gene, the Bcl-2 interacting mediator of cell death (*BIM*). EMT can antagonize the response to targeted therapy in lung cancer by inhibiting *BIM* (27). Furthermore, several studies have demonstrated that alveolar type II cells may be common precursors of both lung adenocarcinoma and SCLC. In this regard, SCLC and adenocarcinoma cells may originate from the same cancer stem cells or progenitor cells (28). In addition, amplification of *MYC* has been observed in the SCLC genome sequencing project. *MYCL1* affects the proliferation of SCLC cells, and *MYCL1* knockdown weakens the proliferation of SCLC cells, indicating that *MYC* plays a driving role in the occurrence of SCLC (29, 30). Transformation to SCLC occurs much less frequently in patients with non-*EGFR* mutations than in patients with *EGFR*-driven NSCLC (31). Genetic testing before treatment confirmed an *EGFR* mutation in Case 1, highlighting the risk of transformation. Patients with *EGFR* exon 19 deletion are also more likely to undergo transformation (26). Another study confirmed that the risk of SCLC transformation is six times higher in patients with concurrent *EGFR*, *TP53*, and *RB1* mutations than in patients without mutations (25). Moreover, Achaete-scute homolog 1 (ASCL1) can interact with the Rb-p53 (by *RB1*, *TP53* code, respectively) axis to promote the growth of adenocarcinoma with a higher degree of invasion (32).

SCLC transformation occurs predominantly in non-smoking Asian female patients with adenocarcinoma with *EGFR*-sensitive mutations (33). In this report, Case 1 was an elderly female with no history of smoking and a primary tumor with histological type of lung adenocarcinoma. The patient was treated with gefitinib, a representative drug of first-generation EGFR-TKIs, and exhibited self-discontinuation behavior during treatment. Offin et al. reported that overall survival (OS) was reduced after discontinuation of EGFR-TKIs compared to that in the normal treatment group (34). Our patient exhibited disease progression after 14 months on gefitinib and was considered resistant to gefitinib. The patient was advised to continue treatment with third-generation EGFR-TKIs. A representative drug, such as ositinib, was recommended for first-line treatment of advanced *EFGR*-mutated NSCLC based on mutations in Thr790Met (35), but the patient refused this recommendation. Transformation to SCLC was confirmed by biopsy after 6 months of continued disease progression. Due to the limited tissue samples available for this biopsy method, the histological type of the entire tumor could not be determined, the possibility that the primary tumor included an SCLC component could not be excluded (19).

In addition, *TP53* and *RB1* mutations may be used as predictors of phenotypic transformation in NSCLC with *EGFR* mutations. *TP53* and *RB1* double mutations are the most common gene mutations in SCLC. In particular, the incidence of *RB* gene inactivation in NSCLC-transformed SCLC is almost 100%. *P53* and *RB* play key roles in cell cycle regulation (36, 37). However, genetic testing was not performed after transformation in Case 1. Hence, there was no evidence for the occurrence of *TP53* and *RB1* mutations in this patient. In addition, the rapid increase in NSE levels and rapid resistance to EGFR-TKIs were suggestive of the transformation of EGFR-positive lung adenocarcinoma to SCLC (38, 39). Indeed, the transformation of lung adenocarcinoma to SCLC is a risk factor when NSE levels are five-fold higher than normal (40). In our case, the patient also experienced a rapid increase in serum NSE levels prior to transformation, and transformation to SCLC was confirmed after secondary biopsy. Any additional treatment for NSCLC should consider the possibility of transformation to SCLC. In this regard, dynamic monitoring of serum NSE levels and genetic detection of *TP53* and *RB* gene mutations can be used to predict disease progression and SCLC transformation during treatment of patients with EGFR-TKIs. These non-invasive techniques allow monitoring of a patient’s response to treatment. Notably, there is evidence supporting repeated biopsies and timely adjustment of treatment strategies after transformation. Several studies have confirmed that pro-gastrin releasing peptide (pro-GRP) can be used as a marker to predict the transformation from lung adenocarcinoma to SCLC (41). Indeed, this marker has higher diagnostic sensitivity and specificity for SCLC compared to NSE (42). For the clinicians, phenotypic transformation of SCLC poses a great challenge, because effective treatment regimens are lacking, and the precise mechanisms underlying transformation remain unclear. With the emergence of acquired resistance to EGFR-TKIs in patients in recent years, immunotherapy is being increasingly promoted as a treatment option for SCLC.

Compared with the phenomenon of small cell transformation after EGFR-TKI resistance, the use of PD-1 inhibitors for transformation is rare and retrospective studies are lacking. We systematically evaluated the literature and reviewed previous case reports of histological transformation in ICI resistance. We identified 15 cases of small cell transformation through immunotherapy from 10 articles. Relevant case information is presented in **Table 1**, which summarizes the clinical and genetic features of these cases. Among cases, 11 were men and 4 were women, with an average age of 69 years. Almost all patients had a history of smoking. The primary tumor types included squamous cell carcinoma, adenocarcinoma, and squamous cell carcinoma with neuroendocrine features. The patients were treated with immunosuppressants at the immune checkpoint, and the PD-1 inhibitors used included nivolumab, pembrolizumab, and sintilimab. The best responses to treatment were partial response, progression of disease, and stable disease. The period from the beginning of immunotherapy to the diagnosis of small cell carcinoma transformation ranged from 4 weeks to approximately 3 years. After detection of small cell transformation, regimens for treating SCLC were selected for follow-up treatment. Based on the available survival data of 11 cases, 9 cases died. The average survival time after small cell transformation was 9 months, and two cases exhibited good clinical therapeutic effects. The specific genomic information of these cases is presented in **Table 1**.

**Table 1:** Case reports of histologic transformation treated with ICIs.

	Reference	Age/sex at NSCLC dx	Smoking history	Histology and clinical staging	Genomic profile	Treatment of ICIs	Best response to ICIs	Histologic Transformation	Treatment for transformation	Life cycles after SCLC dx	Time from ICI start to SCLC dx	Patients outcome
1	Case 2	75 M	40 pack-years	Sq	P40, P63(+), CK5/6(+), TTF-1(-)	Pembrolizumab/15 cycles	PR	SCLC	Pembrolizumab	–	18 months	Clinically well
2	Takuma Imakita (12)	64 M	84 pack-years	Sq; stage IIIa	–	Nivolumab/23 cycles	PR	LCNEC/SCLC	CBDCA/CPT, AMR	–	29 months/33 months	–
		70 M	88 pack-years	Sq; stage IIIa	–	Nivolumab/5 cycles	SD	SCLC	ETP	–	16 months	–
3	Qian Shen (15)	69 M	30 pack-years	Sq; stage IVa	PD-L1≥50%	Sintilimab/4 cycles	PR	SCLC	NA	<1 month	2 months	Died
		71 M	34 pack-years	Sq; stage IIIa	PD-L1 1%	Nivolumab/4 cycles	PD	SCLC	ETP/DDP	5 months	1 month	Died
4	Kartik Sehgal (16)	Mid-60s F	35 pack-years	Sq; stage IV	TP53 mut, CDKN2A R58 mut	Nivolumab/47 cycles	PR	SCLC	CBDCA/ETP/Nivolumab/XRT	14 months	21 months	Died
5	Naoko Miura (17)	65 M	34 pack-years	Ad; stage IVb	EML4-ALK(-), PD-L1(+++)	Pembrolizumab/6 cycles	SD	SCLC	DDP/CPT/AMR	17 months	after 6 cycles of ICI	Died
6	Xiaoyan Si (18)	69 M	48 pack-years	Sq; stage IV	PD-L1≥1%	Pembrolizumab/22 cycles	PR	SCLC	CBDCA/ETP	–	14 months	Lesions decreased
7	Jair Bar (19)	70 F	current	Sq with neuroendocrinefeatures	P40, CK5/6(+), CK56(+), TTF-1(-)	Nivolumab/3 cyclesNivolumab/10 months	PD/SD	SCLC/Mixed neuroendocrine and Sq	Nivolumab	Alive 9 months post SCLC dx; then lost to follow-up	16 months	–
		75 M	>10 pack-years	Sq with neuroendocrinefeatures	P63, CK903(+++), CD56(±), TTF-1(-)	Nivolumab/6 months	PR	SCLC	CBDCA/ETP	13 months	7 months	Died
8	Komugi Okeya (20)	66 M	45 pack-years	Ad; stage IVb	EGFR(+), ALK(-), PD-L1 90-100%	Pembrolizumab/2 cycles	Hyper PD	SCLC	CBDCA/ETP/AMR	4 months	5 weeks	Died
9	Wade T. Iams (21)	67 F	50 pack-years	Ad	–	Nivolumab/36 cycles	PR	SCLC	CBDCA/ETP/TAX	11 months	19 months	Died
		75 F	30 pack-years	Ad	KRAS G12C mut	Nivolumab/33 cycles	SD	SCLC	CBDCA/ETP; Nivolumab/Ipilimumab; CPT	16 months	22 months	Died
10	Nadine Abdallah (22)	65 M	35 pack-years	Ad; stage IVa	EGFR mut, ALK(-)	Nivolumab/5 cycles	PD	SCLC	CBDCA/ETP	–	after 5 cycles of ICI	–
		68 M	Not described	Right lung: SqLeft lung: NSCLC	AE1/AE3(+), TTF-1, CK5/6, P63(-)	Pembrolizumab/CBDCA/PTX, 4 cycles followed by Pembrolizumab, 30 cycles	PR	SCLC	CBDCA/ETP	–	2 years	Clinically well
11	Takuma Imakita (23)	75 M	50 pack-years	NSCLC	EGFR(-), ALK(-)	Nivolumab/3 cycles	SD	SCLC	AMR	1 month	8 weeks	Died

ICIs, immune checkpoint inhibitors; SCLC, small cell lung cancer; NSCLC, non‐small cell lung cancer; dx, dignosis; Sq, squamous cell carcinoma; Ad, adenocarcinoma; PR, partial response; PD, progression of disease; SD, stable disease; NA, Not applicable; CBDCA, carboplatin; ETP, etoposide; CPT, irinotecan; AMR, amrubicin; DDP, Cisplatin; TAX, paclitaxel; XRT, radiotherapy.

Case 2 was a 73-year-old male patient with an initial diagnosis of squamous cell carcinoma of the lung. A review of the literature revealed that the primary tumor was also predominantly squamous lung carcinoma. The mechanism of histological transformation after treatment with PD-1 inhibitors is thought to be similar to that with the use of EGFR-TKIs. In addition to the aforementioned potential mechanisms of transformation including *EMT*, *RB1*, and *Tp53* inactivation; *MYC* amplification; and co-origin cells; another possibility is that the original tumor comprised two histological components (43). However, SCLC with neuroendocrine characteristics may have been missed due to insufficient pathological materials for mixed histological diagnosis. In the process of PD, the SCLC component dominates. With a decrease in the number of treatment-sensitive NSCLC cells, the SCLC components that initially existed in tumors with higher malignancy gradually dominate. Our patient received a carboplatin-paclitaxel combined chemotherapy regimen in addition to ICIs before the occurrence of drug resistance. As such, the influence of other drugs in the combined treatment regimen on histological transformation of the tumor should be considered. In addition, molecular genetic changes in patients during treatment were inadequately monitored.

Based on imaging results, our patient exhibited tumor progression after 15 cycles from the start of immunotherapy. Transformation was confirmed after surgical resection for biopsy, with no *EGFR* mutation or *ALK* translocation after transformation. The antitumor activity of pembrolizumab in patients with PD-L1-positive tumors has been demonstrated in a KEYNOTE-028 multi-cohort study (44). The patient is currently continuing treatment with pembrolizumab. After a thorough evaluation of the lesion, the metastase in the left lower lung that developed in this patient after immunotherapy was considered as oligometastatic. Oligometastasis is a transitional stage between a localized primary tumor and widespread metastases, defined as the presence of one to five metastases from a malignant tumor in a distant organ (45). The oligometastatic phase can occur in a variety of clinical situations: patients with a clear and limited number of metastases at diagnosis; patients with multiple metastases with limited residual disease after systemic therapy, or progression of only one lesion after conventional therapy (oligoprogression), or recurrence of localized lesions after treatment (oligorecurrence) (46). Regular monitoring of lesion progression or metastasis during targeted therapy or immunotherapy can help to detect the emergence of new clinical conditions such as oligometastasis in time. The adoption of aggressive local therapeutic approaches, for example, surgical resection, can then provide opportunities for disease control and good prognosis for patients (47). Researchers noted that surgery provided a better prognostic effect for the oligometastatic stage IV NSCLC. For example, when patients with NSCLC develop extrathoracic distant metastasis, the most common site is the brain, and the mean survival time in the group with and without complete surgical resection of brain metastases was 15.4 and 11.5 months, respectively (P=0.002). With adrenal metastases, the mean survival time was 31.1 and 11.3 months in the surgical and non-surgical resection groups, respectively (P=0.001) (48). These data indicate that surgery appears to provide survival benefit when patients develop oligometastasis during treatment.

Survival prognosis after the occurrence of SCLC transformation is poor due to the rapid progression of SCLC and limited surgical treatment. In patients with progressive disease, the average survival time is 6 months, and survival is correlated with smoking status (49). In our patients, after SCLC transformation, the elderly female patient (Case 1) developed a series of complications leading to rapid deterioration and death due to refusal to continue treatment, while the male patient (Case 2) is currently continuing immunotherapy and is in a good general condition.

## Conclusion

In conclusion, we report two cases exhibiting transformation from NSCLC to SCLC after different treatment regimens. Our findings suggest that tumor cells may transform to a more aggressive histological type once resistance occurs, regardless of treatment with ICIs or EGFR-TKIs. The occurrence of histological transformation may be underscored by genomic mutations. This highlights the importance of monitoring serum tumor markers or repeated biopsy using genome sequence, Furthermore, multi-point sampling detection can conduct at the initial diagnosis to reduce the possibility of missed diagnosis of SCLC and facilitate the discovery of drug resistance mechanisms.

## Data availability statement

The original contributions presented in the study are included in the article/supplementary material. Further inquiries can be directed to the corresponding authors.

## Author contributions

YQ, LC, and LP contributed to conception and design of the study. JD and SZ collected information of case. HL wrote the first draft of the manuscript and organized the data. YQ and NW wrote sections of the manuscript. All authors contributed to the article and approved the submitted version.

## Funding

This work was supported by grants from the National Natural Science Foundation of China (grant no. 81860471); Zhanjiang science and technology development special fund Competitive Allocation Project - key projects of disease prevention and control (2021A05145); Provincial Science and technology special fund (“College items+ task list”) project - special topic of basic and applied research (2021A05236).

## Conflict of interest

The authors declare that the research was conducted in the absence of any commercial or financial relationships that could be construed as a potential conflict of interest.

## Publisher’s note

All claims expressed in this article are solely those of the authors and do not necessarily represent those of their affiliated organizations, or those of the publisher, the editors and the reviewers. Any product that may be evaluated in this article, or claim that may be made by its manufacturer, is not guaranteed or endorsed by the publisher.

## References

[1] Bray F Ferlay J Soerjomataram I Siegel RL Torre LA Jemal A . Global cancer statistics 2018: GLOBOCAN estimates of incidence and mortality worldwide for 36 cancers in 185 countries. CA Cancer J Clin (2018) 68(6):394–424. doi: 10.3322/caac.21492 30207593

[2] Oser MG Niederst MJ Sequist LV Engelman JA . Transformation from non-small-cell lung cancer to small-cell lung cancer: Molecular drivers and cells of origin. Lancet Oncol (2015) 16(4):e165–e72. doi: 10.1016/s1470-2045(14)71180-5 PMC4470698 25846096

[3] Indini A Rijavec E Bareggi C Grossi F . Novel treatment strategies for early-stage lung cancer: the oncologist's perspective. J Thorac Dis (2020) 12(6):3390–8. doi: 10.21037/jtd.2020.02.46 PMC7330760 32642264

[4] Puri S Saltos A Perez B Le X Gray JE . Locally advanced, unresectable non-small cell lung cancer. Curr Oncol Rep (2020) 22(4):31. doi: 10.1007/s11912-020-0882-3 32140986

[5] Ganti AKP Loo BW Bassetti M Blakely C Chiang A D'Amico TA . Small cell lung cancer, version 2.2022, NCCN clinical practice guidelines in oncology. J Natl Compr Canc Netw (2021) 19(12):1441–64. doi: 10.6004/jnccn.2021.0058 PMC10203822 34902832

[6] He J Huang Z Han L Gong Y Xie C . Mechanisms and management of 3rd−generation EGFR−TKI resistance in advanced non−small cell lung cancer (Review). Int J Oncol (2021) 59(5):1–20. doi: 10.3892/ijo.2021.5270 PMC8562388 34558640

[7] Zhang C Lin L Guo X Chen P . Significance of genetic sequencing in patients with lung adenocarcinoma with transformation to small cell lung cancer: a case report and systematic review. Transl Cancer Res (2020) 9(5):3725–33. doi: 10.21037/tcr-19-2291 PMC8798927 35117735

[8] Ettinger DS Wood DE Aggarwal C Aisner DL Akerley W Bauman JR . NCCN guidelines insights: Non-small cell lung cancer, version 1.2020. J Natl Compr Canc Netw (2019) 17(12):1464–72. doi: 10.6004/jnccn.2019.0059 31805526

[9] Garon EB Rizvi NA Hui R Leighl N Balmanoukian AS Eder JP . Pembrolizumab for the treatment of non-small-cell lung cancer. N Engl J Med (2015) 372(21):2018–28. doi: 10.1056/NEJMoa1501824 25891174

[10] Osmani L Askin F Gabrielson E Li QK . Current WHO guidelines and the critical role of immunohistochemical markers in the subclassification of non-small cell lung carcinoma (NSCLC): Moving from targeted therapy to immunotherapy. Semin Cancer Biol (2018) 52(Pt 1):103–9. doi: 10.1016/j.semcancer.2017.11.019 PMC5970946 29183778

[11] Ahmed T Vial MR Ost D Stewart J Hasan MA Grosu HB . Non-small cell lung cancer transdifferentiation into small cell lung cancer: A case series. Lung Cancer. (2018) 122:220–3. doi: 10.1016/j.lungcan.2018.06.024 30032836

[12] Imakita T Fujita K Kanai O Okamura M Hashimoto M Nakatani K . Small cell transformation of non-small cell lung cancer under immunotherapy: Case series and literature review. Thorac Cancer. (2021) 12(22):3062–7. doi: 10.1111/1759-7714.14180 PMC8590890 34622569

[13] Fang L He J Xia J Dong L Zhang X Chai Y . Resistance to epithelial growth factor receptor tyrosine kinase inhibitors in a patient with transformation from lung adenocarcinoma to small cell lung cancer: A case report. Oncol Lett (2017) 14(1):593–8. doi: 10.3892/ol.2017.6229 PMC5494666 28693210

[14] Ren X Cai X Li J Zhang X Yu J Song X . Histological transformation of lung adenocarcinoma to small cell lung cancer with mutant C797S conferring acquired resistance to osimertinib. J Int Med Res (2020) 48(6):1–7. doi: 10.1177/0300060520927918 PMC7328482 32600081

[15] Shen Q Qu J Sheng L Gao Q Zhou J . Case report: Transformation from non-small cell lung cancer to small cell lung cancer during anti-PD-1 therapy: A report of two cases. Front Oncol (2021) 11:619371. doi: 10.3389/fonc.2021.619371 34094904 PMC8176117

[16] Sehgal K Varkaris A Viray H VanderLaan PA Rangachari D Costa DB . Small cell transformation of non-small cell lung cancer on immune checkpoint inhibitors: uncommon or under-recognized? J Immunother Cancer (2020) 8(1):e000697. doi: 10.1136/jitc-2020-000697 32581048 PMC7312456

[17] Miura N Matsubara T Takamori S Haratake N Toyozawa R Yamaguchi M . Histological conversion from adenocarcinoma to small cell carcinoma of the lung after treatment with an immune checkpoint inhibitor: a case report. Oxf Med Case Rep (2020) 2020(4):omaa026. doi: 10.1093/omcr/omaa026 PMC7243716 32477576

[18] Si X You Y Zhang X Wang H Wang M Zhang L . Histologic transformation of lung cancer during pembrolizumab therapy: A case report. Thorac Cancer. (2020) 11(3):793–6. doi: 10.1111/1759-7714.13312 PMC7049493 31944570

[19] Bar J Ofek E Barshack I Gottfried T Zadok O Kamer I . Transformation to small cell lung cancer as a mechanism of resistance to immunotherapy in non-small cell lung cancer. Lung Cancer. (2019) 138:109–15. doi: 10.1016/j.lungcan.2019.09.025 31683093

[20] Okeya K Kawagishi Y Muranaka E Izumida T Tsuji H Takeda S . Hyperprogressive disease in lung cancer with transformation of adenocarcinoma to small-cell carcinoma during pembrolizumab therapy. Intern Med (2019) 58(22):3295–8. doi: 10.2169/internalmedicine.2892-19 PMC6911749 31327828

[21] Iams WT Beckermann KE Almodovar K Hernandez J Vnencak-Jones C Lim LP . Small cell lung cancer transformation as a mechanism of resistance to PD-1 therapy in KRAS-mutant lung adenocarcinoma: A report of two cases. J Thorac Oncol (2019) 14(3):e45–e8. doi: 10.1016/j.jtho.2018.11.031 PMC6382512 30543839

[22] Abdallah N Nagasaka M Abdulfatah E Shi D Wozniak AJ Sukari A . Non-small cell to small cell lung cancer on PD-1 inhibitors: two cases on potential histologic transformation. Lung Cancer (Auckl). (2018) 9:85–90. doi: 10.2147/lctt.S173724 30498383 PMC6207227

[23] Imakita T Fujita K Kanai O Terashima T Mio T . Small cell lung cancer transformation during immunotherapy with nivolumab: A case report. Respir Med Case Rep (2017) 21:52–5. doi: 10.1016/j.rmcr.2017.03.019 PMC5376266 28393006

[24] Yu HA Arcila ME Rekhtman N Sima CS Zakowski MF Pao W . Analysis of tumor specimens at the time of acquired resistance to EGFR-TKI therapy in 155 patients with EGFR-mutant lung cancers. Clin Cancer Res (2013) 19(8):2240–7. doi: 10.1158/1078-0432.Ccr-12-2246 PMC3630270 23470965

[25] Mambetsariev I Arvanitis L Fricke J Pharaon R Baroz AR Afkhami M . Small cell lung cancer transformation following treatment in EGFR-mutated non-small cell lung cancer. J Clin Med (2022) 11(5):1429. doi: 10.3390/jcm11051429 35268520 PMC8911080

[26] Yang H Liu L Zhou C Xiong Y Hu Y Yang N . The clinicopathologic of pulmonary adenocarcinoma transformation to small cell lung cancer. Med (Baltimore). (2019) 98(12):e14893. doi: 10.1097/md.0000000000014893 PMC6708892 30896637

[27] Song KA Niederst MJ Lochmann TL Hata AN Kitai H Ham J . Epithelial-to-Mesenchymal transition antagonizes response to targeted therapies in lung cancer by suppressing BIM. Clin Cancer Res (2018) 24(1):197–208. doi: 10.1158/1078-0432.Ccr-17-1577 29051323 PMC5959009

[28] Jiang Y Shou L Guo Q Bao Y Xu X An S . Small-cell lung cancer transformation from EGFR-mutant adenocarcinoma after EGFR-TKIs resistance: A case report. Med (Baltimore). (2021) 100(32):e26911. doi: 10.1097/md.0000000000026911 PMC8360407 34397927

[29] Peifer M Fernández-Cuesta L Sos ML George J Seidel D Kasper LH . Integrative genome analyses identify key somatic driver mutations of small-cell lung cancer. Nat Genet (2012) 44(10):1104–10. doi: 10.1038/ng.2396 PMC4915822 22941188

[30] Rudin CM Durinck S Stawiski EW Poirier JT Modrusan Z Shames DS . Comprehensive genomic analysis identifies SOX2 as a frequently amplified gene in small-cell lung cancer. Nat Genet (2012) 44(10):1111–6. doi: 10.1038/ng.2405 PMC3557461 22941189

[31] Ferrer L Giaj Levra M Brevet M Antoine M Mazieres J Rossi G . A brief report of transformation from NSCLC to SCLC: Molecular and therapeutic characteristics. J Thorac Oncol (2019) 14(1):130–4. doi: 10.1016/j.jtho.2018.08.2028 30217489

[32] Meder L König K Ozretić L Schultheis AM Ueckeroth F Ade CP . NOTCH, ASCL1, p53 and RB alterations define an alternative pathway driving neuroendocrine and small cell lung carcinomas. Int J Cancer. (2016) 138(4):927–38. doi: 10.1002/ijc.29835 PMC4832386 26340530

[33] Shigematsu H Lin L Takahashi T Nomura M Suzuki M Wistuba II . Clinical and biological features associated with epidermal growth factor receptor gene mutations in lung cancers. J Natl Cancer Inst (2005) 97(5):339–46. doi: 10.1093/jnci/dji055 15741570

[34] Offin M Chan JM Tenet M Rizvi HA Shen R Riely GJ . Concurrent RB1 and TP53 alterations define a subset of EGFR-mutant lung cancers at risk for histologic transformation and inferior clinical outcomes. J Thorac Oncol (2019) 14(10):1784–93. doi: 10.1016/j.jtho.2019.06.002 PMC6764905 31228622

[35] Soria JC Ohe Y Vansteenkiste J Reungwetwattana T Chewaskulyong B Lee KH . Osimertinib in untreated EGFR-mutated advanced non-Small-Cell lung cancer. N Engl J Med (2018) 378(2):113–25. doi: 10.1056/NEJMoa1713137 29151359

[36] Rudin CM Brambilla E Faivre-Finn C Sage J . Small-cell lung cancer. Nat Rev Dis Primers. (2021) 7(1):3. doi: 10.1038/s41572-020-00235-0 33446664 PMC8177722

[37] Oser MG Fonseca R Chakraborty AA Brough R Spektor A Jennings RB . Cells lacking the RB1 tumor suppressor gene are hyperdependent on aurora b kinase for survival. Cancer Discovery (2019) 9(2):230–47. doi: 10.1158/2159-8290.Cd-18-0389 PMC6368871 30373918

[38] Meder L König K Fassunke J Ozretić L Wolf J Merkelbach-Bruse S . Implementing amplicon-based next generation sequencing in the diagnosis of small cell lung carcinoma metastases. Exp Mol Pathol (2015) 99(3):682–6. doi: 10.1016/j.yexmp.2015.11.002 26546837

[39] Niederst MJ Sequist LV Poirier JT Mermel CH Lockerman EL Garcia AR . RB loss in resistant EGFR mutant lung adenocarcinomas that transform to small-cell lung cancer. Nat Commun (2015) 6:6377. doi: 10.1038/ncomms7377 25758528 PMC4357281

[40] Zhang Y Li XY Tang Y Xu Y Guo WH Li YC . Rapid increase of serum neuron specific enolase level and tachyphylaxis of EGFR-tyrosine kinase inhibitor indicate small cell lung cancer transformation from EGFR positive lung adenocarcinoma? Lung Cancer (2013) 81(2):302–5. doi: 10.1016/j.lungcan.2013.04.005 23683536

[41] Liu Y . Small cell lung cancer transformation from EGFR-mutated lung adenocarcinoma: A case report and literatures review. Cancer Biol Ther (2018) 19(6):445–9. doi: 10.1080/15384047.2018.1435222 PMC5927699 29461911

[42] Oya Y Yoshida T Uemura T Murakami Y Inaba Y Hida T . Serum ProGRP and NSE levels predicting small cell lung cancer transformation in a patient with ALK rearrangement-positive non-small cell lung cancer: A case report. Oncol Lett (2018) 16(4):4219–22. doi: 10.3892/ol.2018.9158 PMC6126188 30214557

[43] Ahn S Hwang SH Han J Choi YL Lee SH Ahn JS . Transformation to small cell lung cancer of pulmonary adenocarcinoma: Clinicopathologic analysis of six cases. J Pathol Transl Med (2016) 50(4):258–63. doi: 10.4132/jptm.2016.04.19 PMC4963973 27160687

[44] Ott PA Elez E Hiret S Kim DW Morosky A Saraf S . Pembrolizumab in patients with extensive-stage small-cell lung cancer: Results from the phase ib KEYNOTE-028 study. J Clin Oncol (2017) 35(34):3823–9. doi: 10.1200/jco.2017.72.5069 28813164

[45] Berzenji L Debaenst S Hendriks JMH Yogeswaran SK Lauwers P Van Schil PE . The role of the surgeon in the management of oligometastatic non-small cell lung cancer: a literature review. Transl Lung Cancer Res (2021) 10(7):3409–19. doi: 10.21037/tlcr-21-58 PMC8350094 34430376

[46] Hellman S Weichselbaum RR . Oligometastases. J Clin Oncol (1995) 13(1):8–10. doi: 10.1200/jco.1995.13.1.8 7799047

[47] Novoa NM Varela G Jiménez MF . Surgical management of oligometastatic non-small cell lung cancer. J Thorac Dis (2016) 8(Suppl 11):S895–s900. doi: 10.21037/jtd.2016.08.13 27942412 PMC5124594

[48] Xu Q Wang Y Liu H Meng S Zhou S Xu J . Treatment outcome for patients with primary NSCLC and synchronous solitary metastasis. Clin Transl Oncol (2013) 15(10):802–9. doi: 10.1007/s12094-013-1008-2 23430537

[49] Roca E Gurizzan C Amoroso V Vermi W Ferrari V Berruti A . Outcome of patients with lung adenocarcinoma with transformation to small-cell lung cancer following tyrosine kinase inhibitors treatment: A systematic review and pooled analysis. Cancer Treat Rev (2017) 59:117–22. doi: 10.1016/j.ctrv.2017.07.007 28806542

